# The Distribution of Reniform Nematode (*Rotylenchulus reniformis*) in Cotton Fields in Central Queensland and Population Dynamics in Response to Cropping Regime

**DOI:** 10.3390/pathogens13100888

**Published:** 2024-10-11

**Authors:** Linda J. Smith, Linda Scheikowski, Dinesh Kafle

**Affiliations:** 1 Department of Agriculture and Fisheries, Ecosciences Precinct, 41 Boggo Road, Dutton Park, QLD 4102, Australia; dinesh.kafle@daf.qld.gov.au; 2 Department of Agriculture and Fisheries, Tor Street, Toowoomba, QLD 4350, Australia; linda.scheikowski@daf.qld.gov.au

**Keywords:** crop rotation, nematode management, vertical distribution, *Gossypium hirsutum*, corn, sorghum

## Abstract

Reniform nematode (*Rotylenchulus reniformis*) causes significant yield loss in cotton worldwide. In 2012, its detection in the Dawson-Callide region of Central Queensland prompted extensive surveys of cotton fields. The nematode was confirmed in 68% of sampled fields, with populations ranging from 2 to 3870 *R. reniformis*/200 mL of soil. Soil monitoring revealed increasing populations associated with consecutive cotton crops. However, when corn or sorghum replaced cotton, soil nematode populations significantly decreased. A two-year replicated field trial demonstrated that growing a non-host crop (such as biofumigant sorghum ‘Fumig8tor’, grain sorghum, or corn) significantly reduced nematode populations in the top 15 cm of soil compared to cotton. Unfortunately, when cotton was replanted the following season, nematode populations rebounded regardless of the previous crop. Only the ‘Fumig8tor’-cotton rotation resulted in significantly lower nematode populations than continuous cotton. Vertical soil sampling showed that rotating with a non-host crop significantly reduced nematode densities to a depth of 100 cm compared to cotton. However, when the field was replanted with cotton, nematode populations recovered, unaffected by cropping or soil depth. This study emphasises the importance of monitoring reniform nematodes in cotton soils for early detection and defining distribution patterns within a field, which may improve the effectiveness of management practices. These results suggest that one rotation out of cotton is not sufficient, as populations return to high levels when cotton is grown again. Therefore, two or more rotations out of cotton should be considered to manage this nematode.

## 1. Introduction

*Rotylenchulus reniformis* Linford and Oliveira, 1940 (reniform nematode) can parasitise more than 350 plant species representing 77 plant families [1,2] in tropical, subtropical, and warm temperate regions of the world [1]. Upland cotton (*Gossypium hirsutum* L.) is among the crops most severely affected by reniform nematodes. Upon root infection, the nematode adversely affects plant growth, delays flowering and fruiting times, reduces the number and size of the bolls, and decreases lint quality [3]. In the USA, the reniform nematode has been reported to cause estimated annual losses of approximately 20 million kilogrammes of seed cotton, estimated at US$33 million [3,4,5]. Annual cotton yield loss from reniform nematode reported in the National Cotton Council (NCC) Disease Database ranged from 1.14% to 2.37% in the Cotton Belt region but exceeded 8% in the US Mid-South States, such as Mississippi [6]. More recently, in certain states, the proportion of disease-related loss associated with this nematode is greater than 50% [7]. In Australia on cotton, this destructive pest has only been found in Central Queensland [8]. However, this nematode is currently found on other crops in Queensland, New South Wales, the Northern Territory, and northern parts of Western Australia [9]. The recent detection of reniform nematode on sweet potato in the Lockyer Valley, Queensland [10], raises concern due to its proximity to cotton-growing regions.

The first detection of this plant parasite in Australian cotton was recorded on 11 November 2003, in a single field in Emerald in Central Queensland (CQ) [11]. No further detections were made until an investigation of stunted plants in Theodore, located 250 km south-east of Emerald, led to the identification of *R. reniformis* on 23 November 2012 [12]. A broad and intensive soil survey of cotton fields within the Dawson Callide region of CQ was therefore needed to determine the distribution of reniform nematode to better understand the extent of the infestation and population density. This information could then be used to inform cotton growers of the distribution of this pest on farm and advise on-farm hygiene strategies to minimise the risk of introducing the nematode to clean fields.

One unique trait of the reniform nematode is its spatial distribution in infested fields. Tihohod et al. [13] reported that the reniform nematode has a more uniform distribution in cotton fields than other nematode species. Robinson et al. [14] and Westphal and Smart [15] reported that this nematode is often found relatively deep in the soil profile, and in some cases, with more than 50% of the population living at depths greater than 30 cm. Following cotton, reniform nematode has been detected throughout the soil profile to a depth of 120 cm [16] and as deep as 175 cm [17]. Survival of reniform nematodes at depths well below the cultivation layer can directly affect cotton yields [18] and enable rapid population resurgence into the upper horizons when a host is grown [16,19]. In Australia, cotton is commonly grown on heavy clay Vertosol soils [20]. Few studies have been conducted to understand the movement of nematodes in this soil type. In limited and short-term microcosm experiments, movement of free-living nematodes either up or down in a Vertosol soil collected from a cotton field in New South Wales, Australia, appeared to be restrictive even in the presence of plant roots and moisture [21]. The free-living nematodes may not respond to the presence or absence of plant roots as their dietary requirement is different from that of plant-parasitic nematodes [22]. In another glasshouse experiment, when a Vertosol soil collected from a cotton field in Theodore, Queensland, was inoculated with *R. reniformis* at the base of a 16 cm tall pot, the nematodes moved upwards in response to planting of cotton but did not move upward when either a non-host (sorghum) was planted or the soil was left bare [23]. These results support that the reniform nematode moves upwards in a heavy clay Vertosol cotton soil in response to a host under controlled conditions; however, it is not known how they behave at depth under natural field conditions.

Effective management options for reniform nematode are limited in Australia because there are no resistant cotton cultivars available to growers; however, breeding efforts to develop resistant varieties are ongoing [24]. There are also no nematicides registered for use by Australian cotton growers. Alternatively, crop rotation with resistant or tolerant plant species is recommended, although these crops may not always be economically feasible for growers. Effective management of reniform nematodes with grain sorghum (*Sorghum bicolor*) and corn (*Zea mays*) has been demonstrated and therefore is recommended as rotation crops for cotton [3,25,26,27,28,29]. In the USA, cropping sequences that include one or more of these crops have been shown to significantly lower reniform nematode populations [28]. In Israel, a 90% reduction of reniform was recorded when corn or wheat (*Triticum aestivum*) was the rotation crop [30]. One-year rotations with corn are effective in increasing cotton yields [28,31,32,33]; however, populations of reniform nematodes quickly rebound to pre-rotational crop levels by mid-season. A two-year or longer rotation with corn is recommended, as this can result in nematode populations remaining below current economic thresholds throughout the subsequent cotton crop [29,33]. Even with longer rotations out of cotton, the ability of *R. reniformis* to survive without a host [34,35,36] makes the management of this plant parasite extremely challenging.

Cover crops, also known as green manure crops, are strategically planted to fit into a crop rotation programme and may only be grown for a short period of time. Unlike cash crops that are harvested for sale, cover crops are intentionally grown to incorporate back into the soil. Cover crops provide numerous farming system benefits, such as enhanced soil health through the addition of organic material, fixing additional nitrogen in the soil, promoting beneficial microbial communities, enhancing water infiltration and soil porosity, nutrient scavenging, erosion prevention, and as biofumigants to combat soilborne pathogens [37].

Choosing cover crops that are resistant and non-hosting of plant parasitic nematodes is the most reliable way for these crops to contribute to an Integrated Crop Protection programme [38], as they restrict nematode reproduction by denying them an adequate food source [39]. Some cover crops are allelopathic and kill plant-parasitic nematodes by the production of toxic compounds [39,40,41]. Forage sorghum has been shown to be an effective cover crop to manage plant-parasitic nematodes such as root-knot [40] and reniform [42] by decreasing the infestation potential of soils. In a field study, a cover crop of forage sorghum not only significantly reduced reniform nematode populations in the following cotton crop but also increased cotton yields from plots cultivated with sorghum during the winter compared to a clean fallow [42]. In addition, the leaves and roots of sorghum contain the cyanogenic glucoside dhurrin, which can degrade into hydrogen cyanide (HCN) [43,44], which is known to be toxic to nematodes [45,46,47,48]. In in vitro bioassays, the ovicidal and slow nematicidal effects of dhurrin degradation products against J2 (second-stage juveniles) of the plant parasitic nematodes were reported [49]. While most sorghum species will have some biofumigation activity [38], the only sorghum cultivar bred for its biofumigation properties is ‘Fumig8tor^TM^’ (Pacific Seeds, Toowoomba, Queensland). ‘Fumig8tor’ sorghum is a warm-season crop producing its greatest biomass during summer [38], and as a cover crop was grown as standard practice in the vegetable industry to manage the fungal pathogen *Macrophomina phaseolina* [50].

The objectives of this research were: (1) to understand the distribution and soil population of reniform nematode in the four sub-regions of the Dawson-Callide in CQ; (2) to monitor soil populations over time in fields with different cropping regimes to gain an understanding of population dynamics; and (3) to investigate the potential of non-host crops, such as biofumigation sorghum ‘Fumig8tor’, grain sorghum, and corn, to reduce soil population density of reniform nematode in a replicated strip trial conducted over two seasons on a commercial cotton farm in Theodore in CQ, Australia.

## 2. Materials and Methods

### 2.1. Spatial Distribution of Reniform Nematode in the Theodore District

Following the first detection of reniform nematodes in the Theodore region of Central Queensland on 23 November 2012, 143 fields covering 16 farms in the Theodore South, Theodore East, Theodore West, and Gibber Gunyah growing areas were sampled (cored) to determine the spatial distribution of reniform nematodes within the region. Sampling to a depth of 15 cm was conducted as described in Section 2.4.1. Sampling was undertaken just after harvest, when populations would be at their highest, to increase the potential for detection and enable the comparison of soil populations of fields. Nematodes were extracted from soil samples and counted as described in Section 2.4.2.

### 2.2. Population Dynamics of Reniform Nematode in Response to Cropping Regime

To determine the impact of cotton versus a non-host crop on the soil population of reniform nematode in the top 15 cm, two fields (7 and 8) on a commercial cotton farm in Emerald, Queensland, were sampled post-harvest. In early October 2012, both fields were planted to cotton cultivar ‘Sicot 74BRF’ (Cotton Seed Distributors, Wee Waa, NSW, Australia), which was managed through to harvest as per commercial practice. In September 2013, both fields were planted to grain sorghum hybrid ‘MR Buster’ (Pacific Seeds, Toowoomba, QLD, Australia). The crops headed in January 2014 and then again around 10 May 2014; hence, sorghum grain was harvested twice. Crop residues in field 7 were mulched only, with little soil disturbance. In field 8, residues were not mulched or tilled; they were left standing.

For soil sampling, field 7 was divided into six 10 ha blocks labelled 7A, 7B, 7C, 7D, 7E, and 7F, and field 8 was divided into four 10 ha blocks labelled 8A, 8B, 8C, and 8D. Blocks ran the length of the rows from head ditch to tail drain. Soil cores were taken post-harvest of cotton and grain sorghum on 28 June 2013 and 20 May 2014, respectively, as described below. Reniform populations were estimated as described below.

### 2.3. Reducing Soil Populations of Reniform Nematode with Non-Host Crops (Field Trial)

To investigate the potential of non-host crops to reduce the soil population density of reniform nematode compared to cotton, a replicated blocked strip trial was conducted over two growing seasons on a commercial cotton farm in Theodore in CQ, Australia.

#### 2.3.1. Cotton Growing Season 2014/15

The trial design consisted of four treatments × eight rows × six replicate plots. The field was split into three blocks to account for potential variability in nematode populations due to a strip trial the previous season. The treatments were randomly assigned within each block. Each row was a raised bed 1 m wide and furrow irrigated. Each treatment plot consisted of eight planting rows running the entire length of the field. In the 2014/15 season, treatments included three non-host crops: sorghum biofumigation cultivar ‘Fumig8tor^’^, grain sorghum hybrid ‘MR Buster’ and corn hybrid ‘606’ (Pacific Seeds, Toowoomba, QLD, Australia), and the host crop cotton, cultivar ‘Sicot 74BRF’ (Cotton Seed Distributors, Wee Waa, NSW) as the control treatment. On the 29th and 30th of September 2014, the field trial was marked out, and soil was sampled prior to planting as described below to determine the initial population in the top 15 cm of soil. A total of 100 cores per plot were collected in a zigzag pattern across all eight rows the full length of the field. Nematodes were extracted, and *R. reniformis* was counted as described below to determine the soil population of reniform nematodes.

The trial was planted in early October 2014 and managed by the grower using standard farm practices, including fertilisation, irrigation (flood furrow), and weed management. Biofumigant sorghum was sown at a rate of 25 kg/ha, grown to approximately 1 m tall, then slashed and mulched, leaving the residues on the soil surface. The crop was then irrigated, grown until maturity (25% flowering), then root cut, mulched green, and incorporated.

To determine the impact of a host crop versus a non-host crop on the soil population of *R. reniformis*, sampling of the top 15 cm of soil was conducted as per the protocol described below on the 31 March 2015 after harvest of cotton, biofumigant sorghum ‘Fumig8tor’, grain sorghum, and corn.

#### 2.3.2. Cotton Growing Season 2015/16

To determine the impact of previous crop on soil population of *R. reniformis* in the field when cotton is re-introduced, the field was sown with Bollgard III cotton cultivar ‘Sicot 746B3F’ in late September 2015. The trial was managed by the grower using standard farm practices. Sampling of the top 15 cm of soil was conducted, as described below, on the 2nd of March 2016 after harvest. Nematodes were extracted and *R. reniformis* counted.

### 2.4. Vertical Distribution and Population Dynamics

For successful management of this pest, it is essential to understand the distribution patterns influencing the presence and abundance of the nematode and the factors driving such distribution. With assistance from Dawson Ag Consulting Pty. Ltd. (Theodore, QLD, Australia), the vertical distribution of reniform nematodes was assessed. Soil cores to a depth of 100 cm were taken from each replicate treatment in the non-host rotation trial 50 m in from both the tail drain and head ditch ends of the field in the 2014/15 season. In the 2015/16 season, the field was planted to cotton, and after harvest, each plot was sampled again to a depth of 100 cm, 50 m in from the tail and head drains, as well as midfield. A Christie post driver on a Honda GX35 engine was used to drive a Dig Stick Spurr Soil Probe to a depth of one metre. Once removed, each soil core was divided into three lengths consisting of 0–30 cm, 30–70 cm, and 70–100 cm. Nematodes were extracted from each soil sample and counted as described below; however, the reniform nematode population was calculated per 200 g of oven-dried soil instead of per 200 mL of soil. The mean population of the sampling site (tail-drain and head-ditch in 2015 and tail-drain, midfield, and head-ditch in 2016) for each replicate treatment was reported and used in analyses. The data collected will provide information on the influence of crops on the vertical distribution of reniform nematodes at the end of the season.

#### 2.4.1. Cotton Soil Sampling Protocol

To establish nematode populations and distribution in Theodore fields, each field was subdivided into approximate 10-hectare blocks to avoid biassed results arising from the clumped distribution patterns of nematodes. Soil samples of the heavy clay Vertosol soils were taken in a zigzag pattern, one core per stop, using a 17 mm foot-pushed soil corer to a depth of 15 cm, until a total of 100 soil cores were collected across the 10-ha block. For pre-plant samples, soil was taken after the beds were formed in the proposed planting area from the top of the hill. Post-harvest, samples were taken at a 45° angle, 15 cm deep, and 10 to 12 cm from the planted row, targeting the root zone. If dry topsoil was present, it was scraped off before sampling. The 100 soil cores were placed directly into a bucket, mixed thoroughly, and a subsample of approximately 400 g was double-bagged in a zip-lock bag and clearly labelled. Extraction depends on live nematodes; therefore, samples were kept cool in an esky for transportation to the laboratory for processing.

#### 2.4.2. Nematode Extraction and Counting

Nematodes were retrieved from soil samples using a modified version of an extraction method developed by Whitehead and Hemming [51] that is commonly referred to as the Baermann tray technique. Samples consisting of multiple cores from the subsample were broken apart by hand where possible and mixed uniformly, then a 200-mL subsample of soil was spread on a tissue-covered mesh basket. The basket was then placed in a tray, and water was added to saturate the soil. Trays were incubated inside a cabinet for three days at room temperature. To retrieve the nematodes, the metal sieve containing the soil was removed from trays, and the remaining solution was poured through two fine sieves, one to remove soil debris (150 µm-aperture) and one to collect the nematodes (38 µm-aperture), which were rinsed from the sieve surface with water into a vial. The plant-parasitic reniform nematode *R. reniformis* (and other plant-parasitic nematodes including *Pratylenchus* sp., *Helicotylenchus dihystera*, *Rotylenchus brevicaudatus*, *Paratrichodorus minor*, *Meloidogyne* sp., *Tylenchulus* sp., and *Merlinius brevidens* if present) were identified and counted under a compound microscope at a magnification of 40× and reported as number per 200 mL of soil for samples collected from the top 15 cm of soil only. Following extraction of the samples collected to a depth of 100 cm and divided into three sections (0–30 cm, 30–70 cm, and 70–100 cm), the soil with the tissue paper was dried in an oven at 60 °C for three days, then weighed to obtain nematode densities per gramme of dry soil and reported as a number per 200 g of soil.

### 2.5. Statistical Analysis

Nematode data were transformed [log10(no. nematodes + 1) or √no. nematodes] to standardise the variance, and the data were analysed using statistical analysis software (Genstat 22nd Edition). A one-way or two-way analysis of variance (ANOVA) for a blocked design was used to examine treatment effects. Where there was a significant (*p* ≤ 0.05) difference between means (using the ANOVA output), the Fisher’s Protected Least Significant Difference (LSD) test (*p* ≤ 0.05) was performed to separate means. A paired *t*-test was used to compare the means of reniform nematode populations in the soil post-harvest cotton versus post-harvest sorghum.

## 3. Results

### 3.1. Spatial Distribution of Reniform Nematode in the Dawson-Callide Region

#### 3.1.1. Autumn 2013—Post-Harvest Survey

**Figure 1:**
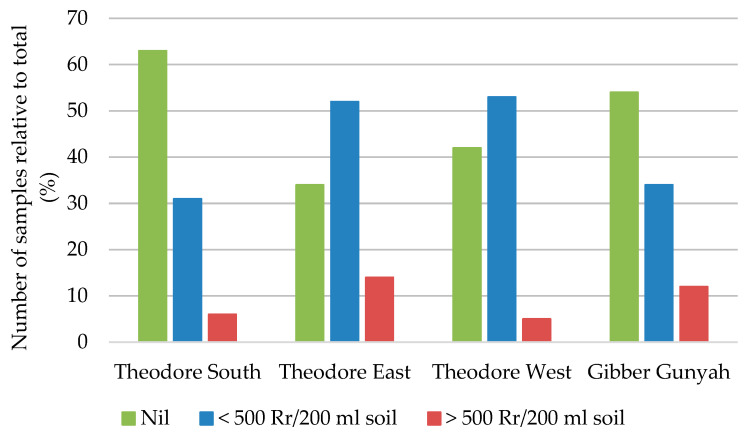
Proportion of soil samples collected post-harvest cotton in 2013 that represent a nil detection of *Rotylenchulus reniformis* (Rr), populations less than 500, between 500 and 1000 nematodes, or greater than 1000 nematodes per 200 mL of soil for each of the four sub-regions in the Dawson-Callide region.

Figure 1 depicts the number of samples (each from 10 ha field sections) associated with nil detections and three broad density categories (<500, 500–1000, and >1000 nematodes/200 mL soil) for the four production areas in the Dawson-Callide region. These density divisions were used to convey some range of the nematode densities measured and are not associated with a particular level of crop damage, as this is not yet understood for the Australian cotton farming system. Internationally, it has been estimated that the economic damage threshold (number of nematodes required to cause a 10% or greater yield loss) is 1000 reniform nematodes/200 mL of a clay loam soil for samples collected post-harvest [52]. Hence, for comparison, the percentage of samples exceeding 1000 nematodes per 200 mL of soil was included.

The reniform nematode was distributed widely throughout the region and was confirmed in 68% of fields sampled. No other plant-parasitic nematodes of cotton were detected. There was a general increase in incidence (a decreasing proportion of nil detection) in order of Theodore South, Gibber Gunyah, Theodore West, and Theodore East. Although incidence in Theodore South was comparatively low, relatively high densities were detected in all sub-regions. In soil samples where the reniform nematode was confirmed, the population density in samples from Theodore South, Theodore East, Theodore West, and Gibber Gunyah ranged from 2–3870, 2–972, 2–2041, and 5–1951 *R. reniformis*/200 mL soil, respectively. Three of the four sub-regions had populations greater than 1000 nematodes/200 mL of soil.

#### 3.1.2. Autumn 2013—Post-Harvest Survey

**Figure 2:**
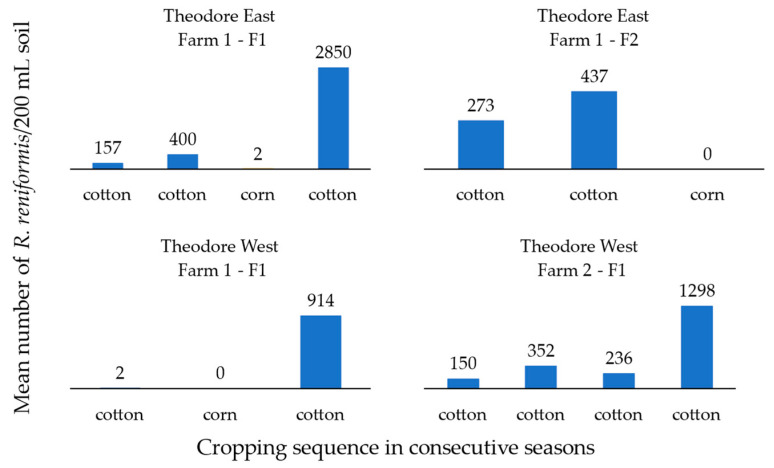
Mean reniform nematode (*Rotylenchulus reniformis*) field population/200 mL of soil in the top 15 cm post-harvest of crop from four fields on three commercial cotton farms in the Theodore region in Queensland. First crop in sequence was grown in the 2012/13 season and included cotton-cotton-corn-cotton, cotton-cotton-corn, cotton-corn-cotton, and continuous cotton.

**Figure 3:**
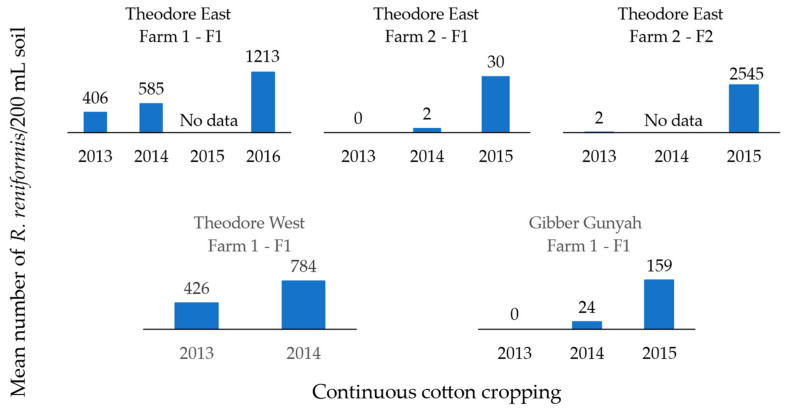
Mean reniform nematode (*Rotylenchulus reniformis*) field population/200 mL of soil in the top 15 cm post-harvest cotton grown back-to-back from five fields on four commercial cotton farms in the Theodore region in Queensland.

The population densities observed after each rotation cycle behaved as expected based on the known host suitability of the different crops (Figure 2 and Figure 3). The mean reniform nematode population densities in soil post-harvest were lowest when corn was included in the cropping sequence, reducing the populations from 400 to 2 and 437 to 0, in fields 1 and 2 on Farm 1 Theodore East, respectively (Figure 2). Soil populations of reniform nematode increased following continuous cotton (Figure 3). The highest soil population of 2850 *R. reniformis*/200 mL of soil was observed in post-harvest cotton, even though the mean soil population of the previous corn crop post-harvest was only 2 *R. reniformis*/200 mL of soil (Theodore East Farm 1—F1). This trend was also observed in a second field (Theodore West Farm 1—F1), where no reniform nematodes were detected after corn in the top 15 cm of soil; however, when planted to cotton in the following season, the post-harvest soil population increased to 914 *R. reniformis*/200 mL of soil (Figure 2).

**Figure 4:**
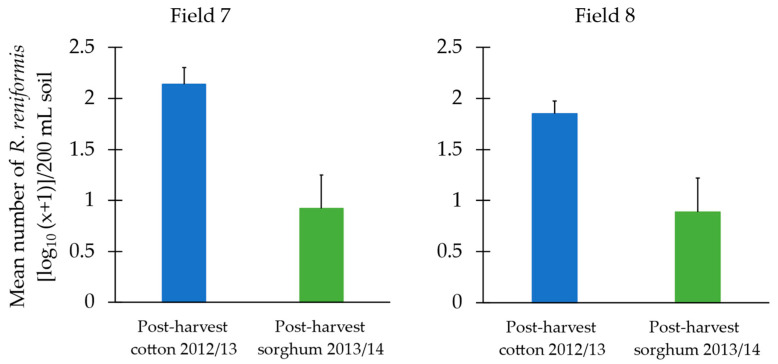
The mean population of *Rotylenchulus reniformis* [log_10_(x + 1)] per 200 mL of soil in the top 15 cm post-harvest following cotton in 2013 and sorghum in 2014 in fields 7 and 8 on a commercial cotton farm in Emerald, Queensland. Error bars represent the standard error of the mean (n = 6 for field 7, n = 4 for field 8).

A paired *t*-test showed that the soil population of reniform nematode in the top 15 cm in field 7 was significantly reduced post-harvest sorghum (M = 0.92, SD = 0.80) in the 2013/14 season compared to post-harvest cotton (M = 2.14, SD = 0.39; t(10) = 3.34, *p* = 0.004) in the previous season (Figure 4). Similarly, for field 8, the soil population of reniform nematode was significantly reduced post-harvest sorghum (M = 1.85, SD = 0.66) in the 2012/13 season compared to post-harvest cotton (M = 1.85, SD = 0.25; t(6) = 2.73, *p* = 0.02) in the previous season (Figure 4).

### 3.2. Reducing Soil Populations of Reniform Nematode with Non-Host Crops

#### 3.2.1. Cotton Growing Season 2014/15

**Figure 5:**
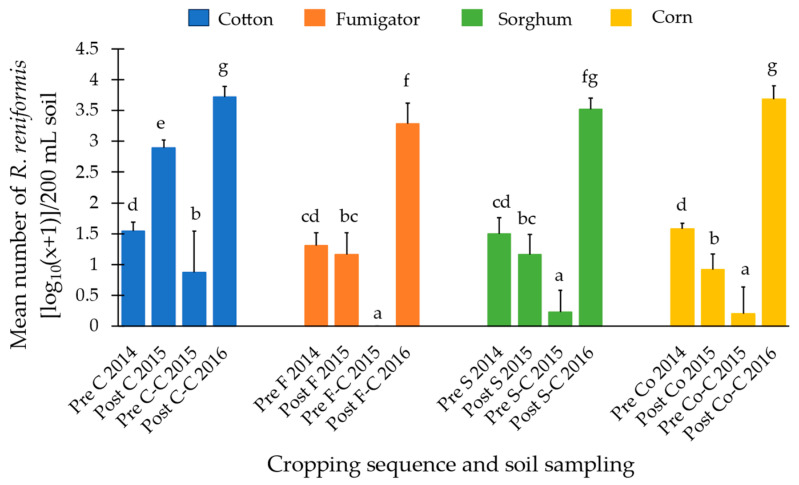
The effect of cotton and non-host summer crops on density of *Rotylenchulus reniformis* [log_10_(x + 1)] per 200 mL of soil in the top 15 cm pre-plant and post-harvest of crop. Pre = Soil sampling pre-planting of crop; Post = Soil sampling post-harvest of crop. C = cotton, F = biofumigation sorghum ‘Fumig8tor’, S = sorghum (grain), Co = corn. Cropping cycle over two seasons: C-C = cotton-cotton, F-C = biofumigation sorghum ‘Fumig8tor’-cotton, S-C = sorghum (grain)-cotton, Co-C = corn-cotton. Error bars represent the standard deviation of the mean (n = 6), and treatments followed by a different letter are significantly different from one another (*p* < 0.001).

The populations of reniform nematode in the top 15 cm of soil pre-plant were consistent across the field, as there were no significant differences between treatment plots. At the end of the season, cotton significantly increased the soil population of reniform nematodes post-harvest of crop compared to the non-host crops, biofumigation sorghum ‘Fumig8tor’, grain sorghum, and corn. There was no significant difference between pre-plant and post-harvest populations when biofumigation sorghum ‘Fumig8tor’ and grain sorghum were grown, whereas corn significantly reduced the soil population of reniform (Figure 5).

#### 3.2.2. Cotton Growing Season 2015/16

All pre-plant soil populations (2015/16 season) following a non-host crop in the 2014/15 season were significantly lower than when cotton had been grown, with no reniform nematodes recovered from biofumigation sorghum ‘Fumig8tor’ (F) plots. However, when the field was oversown to cotton, the F-C treatment (2630 *R. reniformis*/200 mL soil) was the only cropping sequence that significantly reduced the reniform population in the soil post-harvest compared to continuous cotton (5704 *R. reniformis*/200 mL soil). There was no significant difference in soil population of reniform nematode between the F-C and grain sorghum (S-C) (3572 *R. reniformis*/200 mL soil) cropping sequence or between S-C and corn (Co-C) (5422 *R. reniformis*/200 mL soil) cropping sequence. There was, however, a significant increase in the soil population of reniform nematodes following the Co-C compared to the F-C. Regardless of previous crop grown in the 2014/15 season, whether a host or non-host of reniform nematode, when plots were oversown to cotton, soil populations post-harvest were higher in 2016 than in 2015 (Figure 5).

### 3.3. Population Dynamics and Vertical Distribution

#### 3.3.1. Cotton Growing Season 2014/15 (Population Dynamics)

Results from a two-way analysis of variance showed that in 2015 there were significant main effects of crop and sampling depth on mean population density of reniform nematode in the soil at the end of the season. Cotton had a significantly higher population of reniform in the soil post-harvest compared to non-host crops biofumigation sorghum ‘Fumig8tor’, grain sorghum, and corn. The three sampling depths were significantly different from each other, with the highest population detected in the 30–70 cm zone, followed by the top 30 cm of soil, and the lowest population at the depth of 70–100 cm (Table 1).

#### 3.3.2. Cotton Growing Season 2015/16 (Population Dynamics)

In 2016, there was no significant main effect of cropping sequence on reniform nematode populations at the end of the season between cotton-cotton, ‘Fumig8tor’-cotton, and sorghum-cotton. However, the reniform population following a corn-cotton rotation was significantly higher at the end of the season compared to all other rotations. There were no significant main effects of sampling depth on the soil population of reniform nematodes at the end of the season (Table 1).

#### 3.3.3. Cotton Growing Season 2014/15 (Vertical Distribution)

**Figure 6:**
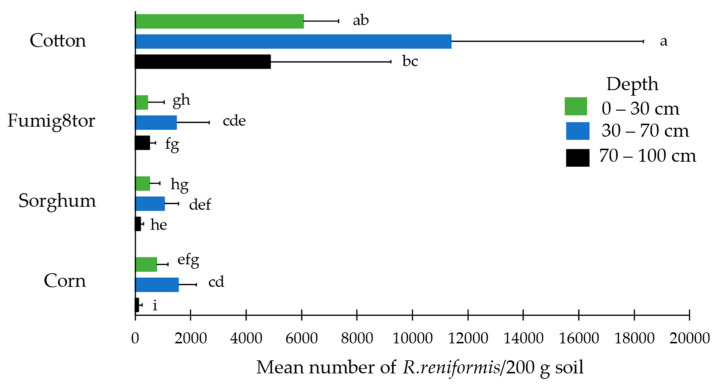
Mean reniform nematode densities (*Rotylenchulus reniformis*/200 g of soil) at three sampling depths for four crops post-harvest in 2015. Error bars represent the standard deviation of the mean (n = 6). The figure represents raw data, but the statistical comparisons are based on the transformed data [log_10_(x + 1)]. Treatments (Crop × Depth) followed by different letters are statistically different from one another (*p* ≤ 0.05).

In 2015 post-harvest, the Crop × Depth interaction effect was significant for mean population density of reniform nematode in the soil (Figure 6). The reniform nematode was found at very high densities at all sampling depths when cotton was grown. The highest average population under cotton was 11,399 *R. reniformis*/200 g of soil at a depth of 30–70 cm, which was significantly higher than the population at 70–100 cm (4872 *R. reniformis*/200 g of soil). There was no significant difference between the population in the top 30 cm (6060 *R. reniformis*/200 g of soil) compared to 30–70 cm.

Rotating with a non-host crop significantly reduced population densities of reniform nematodes at all soil depths measured compared to cotton. For each non-host crop, the population of reniform nematode post-harvest was significantly higher at a soil depth of 30–70 cm, compared to the top 30 cm. All crops, apart from sorghum, had a significantly lower population at a depth of 70–100 cm compared to 30–70 cm (Figure 6).

#### 3.3.4. Cotton Growing Season 2015/16 (Vertical Distribution)

**Figure 7:**
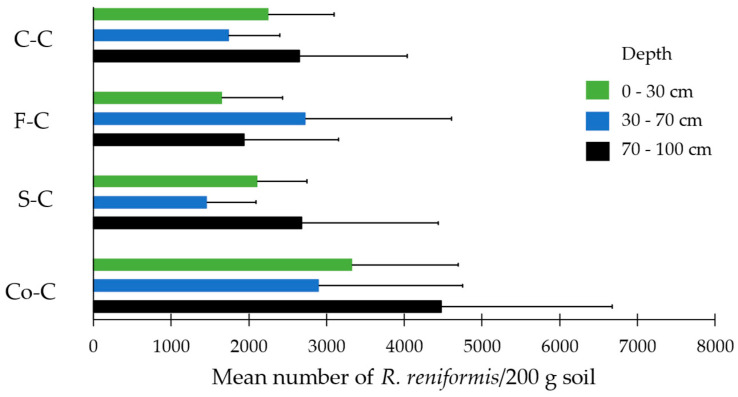
Mean reniform nematode densities (*Rotylenchulus reniformis*/200 g of soil) at three sampling depths for four cropping sequences post-harvest. Cropping cycle over two seasons: C-C = cotton-cotton, F-C = ‘Fumig8tor’-cotton, S-C = sorghum-cotton, Co-C = corn-cotton. Error bars represent the standard deviation of the mean (n = 6). The figure represents raw data, but the statistical comparisons are based on the transformed data [√X]. Treatments (Crop × Depth) were not statistically different from one another (*p* ≤ 0.05).

When planted back to cotton after one growing season with either cotton or a non-host, the population of reniform nematode increased significantly across all plots, with no significant differences between treatments (Crop × Depth interaction). When grown back to cotton, the reduced population of reniform nematode from growing a non-host compared to cotton over one season (Figure 6) was negated (Figure 7).

## 4. Discussion

Reniform nematode is considered a major limiting factor in cotton production [3,53,54]. Therefore, upon confirmation of this plant parasite infecting cotton in the Dawson-Callide region of CQ in 2012, surveys to gather information on its distribution and population density were considered the essential first step. When collecting soil samples for nematode population studies, it is important that the soil sample be truly representative of the area sampled. Improper sampling can lead to poor recommendations and economic losses, which could have been avoided. The only way to ensure that a sample is representative of a field is to collect from many areas around the field rather than from one or two spots [55]. Hence, in this study, 100 soil cores for every 10-ha section of field were collected, combined, and sub-sampled to ensure a representative sample [12]. Results of analyses showed that reniform nematode was widespread in the Dawson-Callide region, being present in all four sub-regions and in 68% of fields sampled with a population ranging from 2 to 3870 *R. reniformis*/200 mL of soil.

Determining nematode population densities to gauge the potential risk to cotton production based on threshold values has been developed overseas, where it was determined 1000 reniform nematodes were required per 200 mL of a clay loam soil post-harvest cotton to cause a 10% or greater yield loss [52]. Based on this international estimate, three of the sub-regions have populations considered to cause economic damage. Although damage thresholds have not been confirmed under local field conditions, it raises concern that populations may have the potential to be economically damaging. In addition, there is anecdotal evidence from local cotton consultants and growers that in some fields where reniform nematodes were detected, yields had been declining and the cause was not able to be determined. Considering the confirmation of reniform nematode and the high populations now determined in-field, this decline may be due to this plant parasite. The damage threshold of reniform nematode for cotton production in Australia warrants investigation.

Reniform nematode is easily introduced into cotton fields via contaminated farming equipment [56,57,58]. Therefore, informing growers of the presence or absence of reniform nematodes provides them with information to implement appropriate strategies, such as farm hygiene protocols to limit movement from infested fields to clean fields on farms, between farms, and to other regions. It was stressed to growers that great care was needed to prevent the spread of reniform nematode into non-infested fields because research has shown that the colonisation of the soil profile can occur quickly and be irreversible and unstoppable once introduced into a field with a susceptible host and adequate moisture [58].

Soil monitoring of fields over several seasons revealed increasing populations of reniform nematodes associated with consecutive cotton crops. These results were expected and concur with findings of Robinson [3], where the practice of monoculture of susceptible cotton cultivars has increased the population of reniform nematodes in the Cotton Belt of the United States. In our study, when either sorghum or corn replaced cotton in monitored fields, soil populations were decreased. These results were anticipated as *R. reniformis* has little or no reproduction on grain crops such as corn or grain sorghum [2], and rotating cotton with these non-host crops has been shown to be an effective management strategy [3,28,29,31]. In the USA, those that have a reniform problem rotate cotton with a non-host routinely before going back into cotton, as this is the only way they can obtain a profitable cotton crop. Benefits, however, may only occur in the first year following the rotation as populations rebound quickly following a one-year rotation [59]. When fields were planted back to cotton in the 2014/15 season after one season of corn, populations not only rebounded, but they were much higher than after cotton in previous seasons. Given these results, a two-year or longer rotation with corn may be needed, as this can result in reniform populations remaining below their current economic thresholds throughout the subsequent cotton crop [29,33].

To further understand two components of crop protection against reniform nematodes: the ability to decrease population densities in the soil and the potential to protect a subsequent susceptible cotton crop in the rotation, a two-year replicated field trial was conducted on a commercial cotton farm. The effects of three non-host crops (a biofumigant forage sorghum ‘Fumig8tor’ as a summer cover crop, grain sorghum, and corn) in a cotton production system were investigated. The three non-host crops significantly reduced nematode populations in the top 15 cm of soil post-harvest compared to cotton; however, when cotton was replanted the following season, nematode populations rebounded regardless of the previous crop. These results concur with our findings from soil monitoring of commercially cropped fields over several seasons.

All pre-plant soil populations collected in the top 15 cm of soil in the 2015/16 season following a non-host crop were significantly lower than when cotton had been grown, supporting reports that rotating with non-host crops is a good strategy to reduce reniform nematode populations [3,28,29,31]. Interestingly, the biofumigant sorghum ‘Fumig8tor’ was the only treatment in which no reniform nematodes were recovered. However, when planted back to cotton, the top 15 cm of soil was repopulated with reniform nematodes. It is known that reniform nematodes can survive at depths well below the cultivation layer due to their ability to enter an ametabolic state during periods of water scarcity (anhydrobiosis) [60,61], and this enables rapid population resurgence into the upper layers when a host is grown [16,19]. Hence the resurgence of reniform populations when rotated back to cotton after ‘Fumig8tor’ may be due to recolonization of the planting zone by the population reservoir surviving in deeper soil layers.

When planted back to cotton, the cover crop ‘Fumig8tor’-cotton rotation was the only cropping sequence that resulted in significantly lower nematode populations than continuous cotton at the end of the season. Sorghum species are known to produce nematicidal cyanides via enzymatic hydrolysis of precursor cyanogenic glycoside/dhurrin [45,46,47,48] and have been reported to reduce reniform populations when grown as a cover crop [42]. In the intact plant tissues, enzymes and substrates are in separate cells: dhurrin in the vacuole of the epidermal cell and catabolic enzymes in the mesophyll cell. It is upon disruption of cellular integrity due to biotic invasion that the enzymic reactions occur, which produce the nematicidal toxin HCN [43,44]. It is theorised that the microbial breakdown of the incorporated biofumigant sorghum over time may have also resulted in the production of HCN over time, which impacted the potential for reniform nematode to infect and reproduce on cotton. This hypothesis is plausible given the findings of Curto et al. [49], who determined using in vitro bioassays that dhurrin degradation products have an ovicidal and slow nematicidal effect on J2 (second-stage juveniles) of plant parasitic nematodes.

To better understand the impact of a non-host on reniform nematode populations throughout the soil profile and their ability to move vertically in the soil and survive at depth in heavy clay Vertosol soil under natural field conditions, populations were monitored at depths of 0 to 30, 30 to 70, and 70 to 100 cm in the crop rotation field trial post-harvest for two seasons. The population density of reniform nematodes on biofumigant sorghum ‘Fumig8tor’, grain sorghum, and corn was significantly lower throughout the soil profile compared to cotton, with the highest population for all crops detected at 30–70 cm. However, when the field was planted with cotton the following season, reniform populations rebounded to significantly higher numbers throughout the soil profile regardless of previous crop. These findings concur with other researchers [14,15] who reported that although following a non-host crop (such as grain sorghum and corn), the reniform population is low throughout the soil profile, high population densities develop under a susceptible host in the crop sequence. Results of this field study also support our previous findings from glasshouse trials in which reniform nematode was determined to move vertically from depth in a Vertosol soil in response to planting of a host plant [23]. Hence, when rotated back to cotton, the reniform nematode was able to move upwards to recolonize the planting zone with the population reservoir in deeper soil layers.

In 2016, there was a significant main effect of the cropping sequence corn-cotton on reniform nematode populations, which was significantly higher at the end of the season compared to all other rotations, including continuous cotton. Research conducted by Hulugalle et al. [62] showed that cotton sown after corn had deeper and more extensive root densities than cotton sown after cotton. Previous research at the same site [63] showed that corn roots were able to proliferate in the wetter and oxygen-poor subsoil because of the high density of aerenchyma tissues that were present in corn roots. Corn rotation introduced into cotton monocultures improved lint yields and is considered a suitable rotation crop for irrigated cotton in a two-crop sequence. It was suggested that the following cotton crop was likely able to use the pores created by the corn roots as preferential pathways to penetrate the soil. Also, the corn roots may have improved subsoil aeration directly through these root pores. Anecdotally, cotton growers claim that cotton following corn grows ‘significantly better’. It is plausible that the better growth and potentially larger root system afforded to cotton from root proliferation in the soil profile of the previous corn crop might then result in higher populations of reniform compared to back-to-back cotton.

## 5. Conclusions

The plant parasitic nematode, *R. reniformis*, is increasingly impacting cotton production in Central Queensland’s Dawson-Callide region. It was evident from monitoring soil populations in fields over several seasons and in a 2-year crop rotation trial that rotations with non-host plants such as grain sorghum and corn, including cover cropping with a biofumigant sorghum, are an effective method to lower soil populations of this nematode in the soil profile at depth. However, further strategies are needed to enhance control, as one rotation with a non-host was not sufficient to protect a subsequent susceptible cotton crop in the rotation. The lower economic returns from alternative crops compared to cotton may deter producers from adopting rotation. Therefore, a focus on developing integrated management solutions, including resistant plant varieties (when available), innovative cropping methods, cover crops, and potential chemical treatments that are economically viable long-term, is needed. This study covers multiple locations, of which environmental variables at these sites might affect nematode populations. Data on soil types, moisture levels, and other relevant environmental factors should be included in future studies to aid our understanding of how external factors impact nematode dynamics and help guide management strategies.

## Figures and Tables

**Table 1:** The effect of crop and sampling depth on mean soil population of reniform nematode (*Rotylenchulus reniformis*/200 g of soil) post-harvest in 2015 and 2016.

Factors	Treatment	2015 *	Treatment **	2016 *
		Mean Population Density of Reniform Nematode (*R. reniformis*/200 g Soil)		Mean Population Density of Reniform Nematode (*R. reniformis*/200 g Soil)
Crop	Cotton	4677 b	C-C	2104 a
	Fumigator	339 a	F-C	1894 a
	Sorghum	229 a	S-C	1900 a
	Corn	269 a	Co-C	3254 b
Depth	0–30 cm	479 B	0–30 cm	2530 A
	30–70 cm	1622 C	30–70 cm	1971 A
	70–100 cm	229 A	70–100 cm	2285 A

* Treatments within the same column followed by a different letter are significantly different (*p* < 0.001). The table represents raw data, but the statistical comparisons are based on the transformed data [log_10_(x + 1)]. ** Cropping sequence: C-C = cotton-cotton, F-C = Fumig8tor-cotton, S-C = sorghum-cotton, Co-C = corn-cotton.

## Data Availability

Data are unavailable due to grower privacy.
